# Ethnic differences in the use of intrapartum epidural analgesia

**DOI:** 10.1186/1472-6963-12-207

**Published:** 2012-07-20

**Authors:** Alberto Jiménez-Puente, Nicolás Benítez-Parejo, Jorge Del Diego-Salas, Francisco Rivas-Ruiz, Claudio Maañón-Di Leo

**Affiliations:** 1 Evaluation Unit, Hospital Costa del Sol, Autovía A7, Km. 187, 29603, Marbella, Spain; 2 CIBER Epidemiología y Salud Pública (CIBERESP), Barcelona, Spain; 3 Research Support Unit, Hospital Costa del Sol, Marbella, Spain; 4 Obstetrics and Gynecology Area, Hospital Costa del Sol, Marbella, Spain; 5 Department of Preventive Medicine, Hospital Virgen de la Victoria, Málaga, Spain

**Keywords:** Epidural analgesia, Delivery, Immigrants, Ethnicity

## Abstract

**Background:**

Obstetric epidural analgesia (EA) is widely applied, but studies have reported that its use may be less extensive among immigrant women or those from minority ethnic groups. Our aim was to examine whether this was the case in our geographic area, which contains an important immigrant population, and if so, to describe the different components of this phenomenon.

**Methods:**

Cross-sectional observational study. Setting: general acute care hospital, located in Marbella, southern Spain. Analysis of computer records of deliveries performed from 2004 to 2010. Comparison of characteristics of deliveries according to the mothers’ geographic origins and of vaginal deliveries noting whether EA was received, using univariate and bivariate statistical analysis and multiple logistic regression (MLR).

**Results:**

A total of 21,034 deliveries were recorded, and 37.4% of these corresponded to immigrant women. EA was provided to 61.1% of the Spanish women and to 51.5% of the immigrants, with important variations according to geographic origin: over 52% of women from other European countries and South America received EA, compared with around 45% of the African women and 37% of the Asian women. These differences persisted in the MLR model after adjusting for the mother's age, type of labor initiation, the weight of the neonate and for single or multiple gestation. With the Spanish patients as the reference category, all the other countries of origin presented lower probabilities of EA use. This was particularly apparent for the patients from Asia (OR 0.38; 95%CI 0.31-0.46), Morocco (OR 0.49; 95%CI 0.43-0.54) and other Africa (OR 0.55; 95%CI 0.37-0.81).

**Conclusions:**

We observed a different use of EA in vaginal deliveries, according to the geographic origin of the women. The explanation for this involves a complex set of factors, depending both on the patient and on the healthcare staff.

## Background

Obstetric epidural analgesia (EA) has been shown to be the most effective method for relieving pain in labor. It enables women to better tolerate pain during labor and delivery, without affecting neonatal results, although there seems to be an association between epidural analgesia and instrumental vaginal delivery [1-4].

Relatively few studies have focused on the characteristics of women receiving obstetric EA [5-13]. One significant factor has been identified as that of greater demand for EA by women with a higher socio-economic and/or educational level [5,8,13,14]. Studies carried out in the USA [5,9,11], Canada [8], Israel [12] and Western Europe [15-17] generally agree that EA is less commonly received by immigrant women and by those from minority ethnic groups. Other studies have examined the association between racial origin and various obstetric results, but without focusing on the use of EA [14-17].

Our catchment area, on the Spanish Mediterranean coast, has undergone considerable economic development in recent decades, mainly based on tourism. Over 30% of the resident population is of immigrant origin, from two main sources: the United Kingdom and other Western European countries, with a socio-economic level similar to or higher than that of the Spanish population; and other areas – Africa (fundamentally, Morocco), South America, Eastern Europe and Asia – with a lower socio-economic level than that of the native Spanish population.

Our aim was to describe the use of EA in deliveries performed at a hospital where the patients are from widely varying geographic areas. A secondary aim was to observe other characteristics of the perinatal assistance received by different ethnic groups, which might influence the use of EA.

## Methods

This cross-sectional observational study was carried out at the Costa del Sol Hospital (Marbella, southern Spain), which is part of the Spanish public healthcare system and caters for a population of almost 400,000, of whom over 30% are registered foreign residents. Over 70% of the births in this area take place within the hospital. The Costa del Sol Research Ethics Committee approved the study.

We analyzed the computer records of all deliveries performed at the hospital between May 2004 and September 2010 for which data were available on the mother's age, place of residence and country of birth, the gestational age, neonatal birth weight, delivery mode and labor type, and the use or otherwise of EA. The database consulted did not provide reliable information about other variables of interest such as fetal position, parity, the mother’s obstetric background or socio-economic level. The women's countries of origin were grouped into 8 large geographic zones: Spain, United Kingdom, other Western Europe, Eastern Europe, Morocco, other Africa, Central and South America and Asia.

A descriptive analysis was made of all the deliveries recorded, with mean and standard deviation for quantitative variables, and of frequency distribution for the categorical ones. A study was then performed of the relation between the use of EA in vaginal deliveries (excluding cesarean sections) and the mother’s geographic origin, her age (with two cut-off points, at 20 and 35 years), neonatal birth weight (as a quantitative variable) and of single or multiple gestation, using the Student t test for continuous variables and the chi square test for categorical ones. The same analysis was also performed using multiple logistic regression (MLR) with the forced inclusion of the variables.

The level of statistical significance was set at 0.05 and 95% confidence intervals (CI) were calculated for the Odds Ratios (OR). The database was constructed using the dbase IV program and analyzed using R statistical software [18].

## Results

A total of 21,034 deliveries were studied. The mothers’ mean age was 31 years, with a standard deviation (SD) of 5.5 years. 3.4% were aged under 20 years, and 16.9% were aged over 35 years. Mean neonatal weight was 3272 g (SD 522 g), 18.5% of deliveries were induced and 1.6% corresponded to multiple gestations.

Immigrant mothers accounted for 37.4% of the deliveries. The women came from 127 different countries, chief among which were Spain, Morocco and the United Kingdom, with 57%, 8% and 5% of deliveries, respectively. Table 1 shows the general characteristics of the deliveries performed. There was an important variation in the performance of cesarean sections according to geographic origin, ranging from 18.2% for the women from the United Kingdom to 32.5% of those from African countries. There were also important variations in the frequency of deliveries to mothers at ages of higher risk, with 4% of the women from Central and South America aged less than 20 years, versus less than 1% of those from Western Europe, excluding Spain.

**Table 1:** Characteristics of deliveries performed, by geographic origin of the mother

**Geographic origin**	**N**	**%**	**% Cesarean**	**% Induced**	**%<20 Years**	**%>35 Years**	**Birth Weight**	**%Multiple Gestation**
Spain	11995	57.0%	20.6%	19.3%	3.7%	16.8%	3230.6 (503.9)	1.7%
Central-South America	2426	11.5%	26.3%	16.2%	4.0%	12.7%	3350.3 (539.2)	1.4%
E Europe	774	3.7%	20.5%	15.9%	3.6%	10.3%	3348.6 (528.6)	0.9%
W Europe	1021	4.9%	20.8%	18.4%	0.9%	23.6%	3292.6 (550.5)	2.0%
United Kingdom	1104	5.2%	18.2%	16.9%	2.7%	26.1%	3346.5 (589.0)	1.8%
Other Africa	154	0.7%	32.5%	20.1%	1.3%	19.5%	3227.4 (561.1)	1.9%
Morocco	1771	8.4%	20.1%	19.0%	3.2%	17.1%	3382.5 (520.3)	1.2%
Asia	572	2.7%	19.9%	13.6%	1.9%	15.6%	3264.9 (514.1)	0.9%
Others/Unknown	1217	5.8%	21.9%	20.0%	2.9%	16.3%	3242.2 (515.3)	2.1%
**Total**	21034	100%	21.2%	18.5%	3.4%	16.9%	3272.2 (521.9)	1.6%

Weight in grammes expressed as means (standard deviation).

Table 2 shows the bivariate analysis for the study variables according to whether or not EA was supplied. The use of EA in vaginal deliveries was more frequent among the younger women, when neonatal birth weight was greater and for multiple gestations. 61.1% of the Spanish women and 51.5% of the immigrants received EA, with important variations according to geographic origin: thus, over 52% of the other Europeans and the South Americans received EA, compared to about 45% of the Africans (including Moroccans) and 37% of the Asian patients.

**Table 2:** Characteristics of the mothers and neonates, by use of intrapartum epidural analgesia in vaginal deliveries

	**No use of epidural n**	**%**	**Use of epidural n**	**%**	**Bivariate P value**	**MLR Odds Ratios (95% CI)**
Number of deliveries	7040	42.5	9526	57.5		
Age						
20-35 years	5530	41.5	7791	58.5	<0.001	1
<20 years	213	34.1	411	65.9		1.37(1.15-1.63)
>35 years	1297	49.5	1324	50.5		0.70 (0.64-0.76)
Birth weight (SD)	3279.9 (484.9)		3301.0 (475.6)		0.005	1.0002 (1.0001-1.0003)
Multiple gestation	43	31.6	93	68.4	0.013	1.71 (1.17-2.49)
Labor type						
Spontaneous	6295	45.8	7446	54.2	<0.001	1
Induction	734	26.3	2052	73.7		2.44 (2.22-2.67)
Geographic origin						
Spain	3707	38.9	5818	61.1	<0.001	1
Central-South America	765	42.8	1022	57.2		0.84 (0.75-0.93)
E Europe	261	42.4	354	57.6		0.84 (0.71-1.00)
W Europe	358	44.3	451	55.7		0.82 (0.71-0.95)
United Kingdom	435	48.2	468	51.8		0.70 (0.61-0.80)
Other Africa	57	54.8	47	45.2		0.54 (0.36-0.80)
Morocco	794	56.1	621	43.9		0.48 (0.42-0.53)
Asia	287	62.7	171	37.3		0.39 (0.32-0.47)

Weight in grammes (g).

Bivariate analysis: chi square test for categoric quantitative and categorical variables and Student’s t test for continuous ones.

MLR model: -2LL initial = 22478.6; -2LL final = 21473.8; Chi-square = 734.8; p < 0.001.

Hosmer and Lemeshow Goodness-of-Fit Test: p = 0.17.

Bivariate and multiple logistic regression (MLR) analysis.

**Figure 1:**
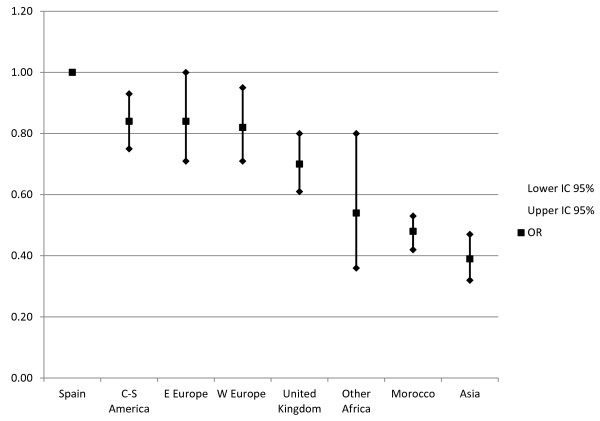
Epidural analgesia used in vaginal deliveries, odds ratios adjusted for mothers’ geographic origin.

Table 2 shows the MLR model for the relation between the provision of EA in vaginal deliveries and the patient's geographic origin, in a model adjusted for mother's age, neonatal weight and single/multiple gestation. Figure 1 shows that, with the Spanish patients as the reference category, all the other countries of origin presented lower probabilities of EA use. This was particularly apparent for the patients from Asia (OR = 0.38; 95%CI 0.31-0.46), Morocco (OR = 0.49; 95%CI 0.43-0.54) and other Africa (OR = 0.55; 95%CI 0.37-0.81).

## Discussion

Our study highlights the relation between the women's geographic origin and a series of characteristics related to the delivery: percentage of cesarean sections, high risk ages, neonatal birth weight and single/multiple gestation. We also describe the relation between the women's geographic origin and the use of EA in vaginal deliveries, this being more common among the Spanish women than among those from elsewhere, especially Africa and Asia.

Forty two percent of the women gave birth without EA and this percentage rose to 48.5% among women from abroad and to over 50% and 60% among those from Africa and Asia, respectively. Some studies have focused on women's preferences for EA [5-7,9,12], others on its recommendation by healthcare staff [12], while the majority, including our own, on the real use of EA [5,8,9,11,15-17]. Note that the use of EA depends on the interaction among three groups of factors: the women's preferences and knowledge, the recommendations made to them and the real availability of EA at the moment in question. For example, a study carried out in France reported that the most common reason for not receiving EA was that the delivery took place too quickly (44%), while only in 37% of the cases was this due to the woman's own decision [14] and another study performed in Canada reported a lower use of EA in women living further away from the hospital [19].

Our results largely concur with those reported by another study carried out in Spain, in a geographic area where immigration is motivated fundamentally by economic concerns, which described the use of EA in 75% of deliveries to Spanish women, in 68% of those to Latin American women, in 49% of those to women from Eastern Europe and in 52% of those to women from Morocco [15].

Other studies have identified a greater demand for EA from women with a higher socio-economic or educational background [5,8,12-14]. Less information is available about the influence of parity [10,12-14], residence in rural or urban environments [11], the woman’s age [12], labor type [5], a more traditional mentality [6,12], concern about the secondary effects of EA [10] or the couple's preferences [7]. On the other hand, opinions are unanimous that EA is less commonly used among immigrant women and those from minority ethnic groups [8,9,11,12,15-17].

With respect to possible explanations for the lesser use of EA by immigrant women, these can be summarized in three groups of potentially influential factors:

1. Less demand for EA by immigrant women.

The geographic origin of mothers could be related to their socio-economic and educational level, such that the latter factor is what really influences the use of EA, rather than the ethnic component [20,21]. Countering this argument is the fact that women from the United Kingdom and from other Western European countries, who do not present special socio-economic differences from the Spanish population, present significantly lower rates of EA use. In the USA, it has been reported that black [9] and Hispanic [5,9] women tend to make less use of EA, after controlling for clinical and economic factors although in our case, socio-economic information was not available.In this respect, it should be noted that the Spanish public health system provides universal, free health care, and so the lack of financial resources should not be any obstacle to accessing health services. In fact, it has been shown that the use of emergency and hospitalization services is independent of users’ socio-economic level [22]. Deliveries in this area that are performed at clinics or centres other than our own hospital are not, in general, considered to represent situations of special clinical risk, but mostly involve women who have private health insurance and who prefer to give birth in such clinics.Foreign women might have less information about the availability of EA, basically due to their lack of familiarity with the local language, lower degree of involvement with the healthcare system, limited participation in antenatal education, or apprehension regarding an unknown medical technique. Various studies, carried out both in Spain and in other European countries, have reported that immigrant women attend antenatal sessions later and less frequently than do local women [15-17].The geographic origin of the women could also lead to communication difficulties, both in expressing their wishes and in understanding the provision of EA, at the moment of delivery. This difficulty would be much less apparent in the case of South American patients, who speak Spanish, and in fact the latter present a significantly higher use of EA than do patients from other countries that are less economically developed than Spain.Our hospital has a volunteer interpreter service covering most European languages and the majority of the staff can maintain basic communication in English. Moreover, the Andalusian Health Service has a phone-based simultaneous translation service in over 60 languages. However, the urgency of many deliveries and their unpredictable timing makes it impossible to guarantee that in all cases there will be a suitable interpretation service, especially in non-European languages.With regard to the socioeconomic status of the country of origin, the culture of the native country also has much to do with EA use. For example, in the United Kingdom, women prefer to use nitrous oxide (entonox) for labor analgesia as opposed to EA [23].Finally, it has been reported that women with more traditional mentalities may consider pain to be inherent to the childbirth process and for this reason they reject EA [6,12]. This “traditional" mentality may be encountered more frequently among women from financially less-developed countries, although significant differences in pain acceptance have been described too in neighboring countries such as Belgium and the Netherlands [24].

2. Healthcare staff are less likely to offer EA to immigrant women:

The existence of variations in medical practice has been well known since the 1970s, when Wennberg first highlighted this phenomenon [25-27]. Thus, patients with similar pathologies may receive differing treatment, according to the criteria of the professional attending them. Many reports have highlighted the influence of factors pertaining to the medical staff, including race, in the recommendations made [28,29]. In the case of pain relief, diverse analgesic treatments have been offered depending on the patients’ gender and racial characteristics [30,31].The question as to whether medical staff provide less EA to immigrant women because it is less frequently requested or vice versa remains to be resolved [12]. Moreover, it has been suggested that if a woman describes her pain in the same cultural way as the medical professional is accustomed to seeing it, then EA is more likely to be recommended [11].

3. Other factors

Reports have described interracial physiological differences in the course of labor and delivery, with shorter durations for black than for white women, and for white than for Asian women [32]. Inter-ethnic differences in pain perception have also been described [30]. The different rate of cesarean sections observed among the different nationalities might provoke selection bias in the application of EA, as our study of the ethnic component was limited to vaginal deliveries. Finally, if immigrant women attend hospital at more advanced phases of labor, this could also discourage the use of EA.

Among other limitations, our study was a cross-sectional one, the design of which did not allow causal relations to be determined between the explanatory and the outcome variables. Moreover, we lacked some adjustment variables that could have been important, such as parity. In this respect, it is well known that primiparous women are more likely to require EA [10,12,14] and first generation immigrants are perhaps more likely to be multiparous. The deliveries resolved by cesarean section were excluded from our analysis as it was not possible to distinguish whether a given delivery aided with EA concluded in the performance of a cesarean section or whether on the contrary the cesarean section was performed using EA. We took the patients’ country of birth as an approximation of their ethnic origin, but recognise that some misclassification may have occurred. Finally, the geographic origins of the women were classified into large groups, within each of which there may have been significant heterogeneity.

## Conclusions

Studies carried out in different countries generally agree that intrapartum epidural analgesia is less commonly received by immigrant women and by those from minority ethnic groups. In our catchment area, on the Spanish Mediterranean coast, with an important percentage of population of immigrant origin, we too have observed a lower use of epidural analgesia by immigrant women in vaginal deliveries.

Our results highlight the importance of the communication factor, as women from Western European countries with a socioeconomic status similar to that prevailing in Spain have lower rates of use. There also appears to be a socioeconomic component, as women from South America, who speak Spanish, present a lower degree of epidural use although significantly higher than that observed for women from areas that are less economically developed, like Africa and Asia. Specific interventions would be necessary to ensure that the mother’s geographic origin is not a barrier to access to epidural analgesia during labor.

## Competing interests

The authors declare that they have no competing interests.

## Authors' contributions

AJP designed the study, organised the data collection and formulated the research questions, he drafted the manuscript; CMD contributed to conceptualizing the study, writing the grant proposal and reviewed the manuscript. NBP, JDS, FRR contributed to the analysis of the data and critically reviewed draft versions of the manuscript. All authors contributed to the development of this manuscript. All authors read and approved the final manuscript.

## Authors' information

AJP, M.D., Ph.D. is Responsible of the Evaluation Unit in the Costa del Sol Hospital (Marbella, Spain) and researcher of the CIBER Epidemiología y Salud Pública (CIBERESP), Spain. JDS, M.D. is a Resident Physician of the Department of Preventive Medicine in the Virgen de la Victoria Hospital (Málaga, Spain); NBP, B.Math. and FRR, D.Sc. are members of the Research Support Unit of the Hospital Costa del Sol (Marbella, Spain) and researchers of the CIBER Epidemiología y Salud Pública (CIBERESP), Spain; CML, M.D. is Director of the Obstetrics and Gynecology Area of the Costa del Sol Hospital (Marbella, Spain).

## Pre-publication history

The pre-publication history for this paper can be accessed here:

http://www.biomedcentral.com/1472-6963/12/207/prepub
